# Pilot study of implementing the Shared Healthcare Actions & Reflections Electronic systems in Survivorship (SHARE‐S) program in coordination with clinical care

**DOI:** 10.1002/cam4.5965

**Published:** 2023-04-25

**Authors:** Stephanie J. Sohl, Rajani S. Sadasivam, Carol Kittel, Emily V. Dressler, Stacy Wentworth, Kavitha Balakrishnan, Kathryn E. Weaver, Rebecca Ann Dellinger, Nicole Puccinelli‐Ortega, Sarah L. Cutrona, Kristie L. Foley, Thomas Houston

**Affiliations:** ^1^ Wake Forest University School of Medicine Winston‐Salem North Carolina USA; ^2^ Atrium Health Wake Forest Baptist Comprehensive Cancer Center Winston‐Salem North Carolina USA; ^3^ University of Massachusetts T.H. Chan Medical School Worcester Massachusetts USA; ^4^ Center for Healthcare Organization and Implementation Research VA Bedford Healthcare System Bedford Massachusetts USA

**Keywords:** cancer survivor, health behavior, health coaching, survivorship care planning

## Abstract

**Introduction:**

Initial cancer survivorship care planning efforts focused on information sharing demonstrated limited impact on patient health outcomes. We designed the Shared Healthcare Actions & Reflections Electronic Systems in survivorship (SHARE‐S) program to enhance survivorship guideline implementation by transitioning some effort from clinicians to technology and patients through supporting health self‐management (e.g., healthy lifestyles).

**Methods:**

We conducted a single‐group hybrid implementation‐effectiveness pilot study. SHARE‐S incorporated three strategies: (1) e‐referral from the clinical team for patient engagement, (2) three health self‐management coach calls, and (3) text messages to enhance coaching. Our primary implementation measure was the proportion of patients e‐referred who enrolled (target >30%). Secondary implementation measures assessed patient engagement. We also measured effectiveness by describing changes in patient health outcomes.

**Results:**

Of the 118 cancer survivor patients e‐referred, 40 engaged in SHARE‐S (proportion enrolled = 34%). Participants had a mean age of 57.4 years (SD = 15.7), 73% were female, 23% were Black/African American, and 5 (12.5%) were from a rural location. Patient‐level adherence to coach calls was >90%. Changes from baseline to follow‐up showed at least a small effect (Cohen's *d* = 0.2) for improvements in: mindful attention, alcohol use, physical activity, fruit and vegetable intake, days of mindfulness practice, depressive symptoms, ability to participate in social roles and activities, cancer‐specific quality of life, benefits of having cancer, and positive feelings.

**Conclusion:**

The SHARE‐S program successfully engaged cancer survivor patients. Once enrolled, patients showed promising improvements in health outcomes. Supporting patient self‐management is an important component of optimizing delivery of cancer survivorship care.

## Abstract 2

We designed the Shared Healthcare Actions & Reflections Electronic Systems in survivorship (SHARE‐S) program to enhance survivorship guideline implementation by transitioning some effort from clinicians to technology and patients through supporting health self‐management (e.g., healthy lifestyles). This single‐group hybrid implementation‐effectiveness pilot study demonstrated that the SHARE‐S program successfully engaged cancer survivor patients and that they showed promising improvements in health outcomes. Supporting patient self‐management is an important component of optimizing delivery of cancer survivorship care.

## 1 INTRODUCTION

As the number of people living after cancer diagnosis is increasing, there is a shift in perspective from primarily treating cancer as a terminal to a chronic illness.^1^ This shift increases the need for cancer survivors to become more actively engaged with healthcare providers to optimize their own health outcomes.^2^, ^3^ Survivorship care planning (SCP) has the potential to enhance communication among clinical teams and empower patients and families. Implementation of SCP clinical guidelines initially focused on providing care plan documents, but this information sharing approach focused on the clinical team has demonstrated limited efficacy for improving patient health outcomes in spite of the time investment by clinicians.^4^, ^5^, ^6^ SCP is more than creating a one‐time written document. Recommended next steps for SCP research include enhancing information technology support,^7^ viewing SCP as an opportunity to facilitate patient engagement and support of self‐management (e.g., healthy lifestyles, symptom management), and assessing SCP using implementation studies that consider the clinical context.^4^, ^6^, ^7^, ^8^, ^9^, ^10^

Recent evidence supports that SCP is more efficacious when derived from shared decision making (e.g., patient‐centered or preference‐sensitive) versus provider‐driven processes.^9^, ^11^ These SCP studies were based on the Chronic Care Model (CCM) in the context of transitioning from acute active cancer treatment to survivorship chronic condition management.^9^, ^11^ However, these studies were limited since the delivery model did not include follow‐up contact, which is typically part of the CCM.^9^ The CCM posits that self‐management support is a key component of high‐quality chronic illness care.^12^ Self‐management support includes collaborating with patients to ensure they have the information and skills they need to be actively engaged in the process of their care, which leads to improved health outcomes.^12^ An eHealth enhanced version of the CCM proposes that electronic tools can further support productive patient‐provider interactions.^13^ One method for supporting patients' engagement in their care is to use a patient‐centered communication style and behavioral change strategies (i.e., self‐management coaching).^14^

Current attempts to complete the complex process of SCP within the context of a single clinical visit present challenges (time constraints; healthcare provider may have limited knowledge of effective shared goal‐setting techniques; patients' ability and willingness to commit to goals at the time of the visit).^15^ Visioning reports, including *Crossing the Quality Chasm* ^16^ call for transforming medicine from episodic, in‐person care to the provision of continuous, coordinated care delivery. Technology‐facilitated spaced education (education provided at spaced intervals rather than provided in a condensed format all at once) can offload some of the implementation effort of guideline‐concordant cancer SCP from clinical teams, and give patients *more time* to carefully engage in and consider their healthcare goals.^17^, ^18^, ^19^ Further, although initial survivorship care planning studies aimed to reach participants immediately following treatment, more recent studies with an expanded focus on self‐management have extended that time period to when survivors' acute transition stress may be reduced.^9^ Care plans are intended to be updated over time to accommodate dynamic health needs.^2^, ^6^, ^20^, ^21^ In sum, survivorship care planning can be viewed as an ongoing process.

Our SCP implementation program (SHARE‐S) built upon the above‐cited successful SCP studies^9^, ^11^ and aimed to enhance SCP implementation by increasing ongoing patient engagement. SHARE‐S had three main components: (1) an electronic referral from the clinical team (e‐referral), (2) longitudinal health self‐management coach calls, and (3) text messages to support the coaching process. Our primary objective was to evaluate how successfully SHARE‐S could be implemented into clinical care as characterized by rates of enrollment (a prior enrollment target was that >30% of patients e‐referred to SHARE‐S would enroll). Our secondary objectives included describing other indicators of preliminary implementation success and effectiveness as assessed by patient health outcomes to inform future studies.

## 2 METHODS

### 2.1 Study design

After formative work, we conducted a single‐group pre‐post evaluation hybrid implementation‐effectiveness pilot in collaboration with two Atrium Health Wake Forest Baptist (AHWFB) cancer survivorship programs: one from the Comprehensive Cancer Center and an affiliated Cancer Center. The overall purpose of this pilot was to test the feasibility of implementing and evaluating SHARE‐S in coordination with clinical care. Study procedures were approved by the Wake Forest University Health Sciences Internal Review Board (IRB00064683) and registered with ClinicalTrials.gov (NCT04337203).

### 2.2 Procedures: clinic‐level

The survivorship programs at both cancer centers were under the same leadership. The following procedures were informed by engaging multi‐level stakeholders through individual qualitative interviews conducted with advisory panel members (i.e., health coaches *n* = 2, healthcare providers *n* = 2, cancer survivor patients *n* = 2^22^). Interviews were designed to gather feedback on clinical processes and technology development specific to implementation of the proposed intervention (please see Table S1). For example, this feedback led us to inform participants that responses to the text messaging system were not monitored and to create further links to integrate the guidebook and text messages that supported the coaching intervention. We also iteratively assessed progress and incorporated other suggestions from our continued communication with stakeholders throughout the study. Therefore, we planned for and allowed clinic‐level contextual modifications to refine how the core implementation strategies were delivered over time (e.g., personnel who delivered them).^23^ Decisions about modifications were made in collaboration with the research and clinical teams.

We asked clinic staff to use an e‐referral tool (eRefer) as a part of standard care to notify our study team of patients with an upcoming or recently completed survivorship care planning visit. The eRefer online portal was developed for a prior study.^24^ It allowed potential participants to provide their email and/or cell phone number to a member of the clinic team. For this study we required the use of cell phone numbers only, in order to be more accessible to patients with limited home technology. By providing their contact information, the patients verbally agreed to be sent the study invitation message and contacted by the study team. Referrers were asked to provide only a very brief description of the program.

### 2.3 Procedures: patient‐level

#### 2.3.1 Participants

After receiving an e‐referral with the patient's contact information, the study team contacted patients remotely (e.g., telephone, email), provided further study information, and answered questions to determine willingness to participate. The following inclusion criteria were required for participation: (1) Adults ≥18 years of age; (2) documented or planned cancer survivorship visit; (3) having a working, text‐enabled phone; (4) cognitively able to complete study procedures, as judged by the study team; and (5) able to understand, read and write English. Informed consent was signed remotely through REDCap (Research Electronic Data Capture) or paper mail. Survivorship visits were part of standard care, thus the decision about who qualified for this study was partially based on provider discretion. Each cancer‐specific treatment team determined the appropriate time for survivorship referral based on consensus and national guidelines. In general, patients at low risk for recurrence who had completed definitive therapy were referred for permanent follow‐up in the Survivorship Clinic. In cases where the patient continued on therapy (e.g., breast patients on Herceptin, prostate patients on hormone deprivation), patients were offered a one‐time survivorship visit and they went back to follow‐up with their primary oncologist. We initially planned to start the first coaching call and round of text messages in preparation for an initial survivorship visit and modified the inclusion criteria after engaging the second clinic site to also allow for referrals after any survivorship visit.

#### 2.3.2 Health self‐management coach calls

After baseline procedures were complete, the study coordinator scheduled the first of three health self‐management coach calls and gave participants a paper or electronic (upon request) copy of a Personal Health Journey Guidebook. This booklet summarized recommendations from NCCN Clinical Practice Guidelines for Survivorship with a focus on healthy lifestyles^21^ and facilitated the coaching process. Participants were asked to review the guide before their first coaching call, and to take notes using the pages provided.

Three coaching calls were completed by telephone and spaced approximately 3 weeks apart. The first coaching call was up to 60 min and the two subsequent coaching calls were designed to be 30 min each. Each call was recorded, 20% were selected for treatment fidelity review by an independent observer who used a checklist,^25^ and the coaching team (SJS, observer, and coaches) met six times during the study to refine the coaching sessions. Study coaches had completed health‐related master's degrees, were graduates of a National Board Certified Health & Wellness Coach (NBC‐HWC) approved training program, and the primary coach had completed NBC‐HWC certification. All coaches received initial study‐specific training and participated in meetings where feedback was provided by the independent NBC‐HWC certified fidelity observer.

Coaches engaged participants in the following behavior change techniques that were adopted as implementation strategies to: (1) prime patients to be active participants in their care and (2) enhance survivors' uptake and adherence to the SCP guidelines relevant to self‐management (e.g., healthy lifestyles)^26^: Shaping knowledge (overview of SCP), identity (values), regulation (reduce negative emotion with mindfulness—skill), goals and planning (goal setting, action planning, problem solving, review goals), feedback and monitoring (plan for self‐monitoring of behavior), comparison of outcomes (e.g., imagining/vision of future optimal health), and social support (emotional).^27^, ^28^, ^29^

Coaches supported autonomy by providing a range of general topics for guiding creation of personalized health goals, which were adapted from another telephone lifestyle coaching study as informed by our preliminary work: (1) Eat wisely; (2) Be physically active; (3) Be tobacco‐free/limit alcohol; (4) Strengthen social connections; (5) Restore (e.g., manage Stress); (6) Get adequate rest; (7) Engage in preventive care; (8) Other personal development (e.g., spiritual, work, finance).^30^, ^31^ That is, patient‐selected goals were open‐ended and personalized to realistically fit into their life context. Health coaching included mindfulness as a form of self‐regulation^28^, ^29^ and to enhance autonomy support.^14^

#### 2.3.3 Text messages

After the first coach call, the participant's information was placed in an automatic text‐messaging system. Text messages were sent once daily for 3 weeks after the first (reflect and prepare texts) coach call and once daily for the first 2 weeks following the second (goal support texts) coach call. One additional follow‐up text was sent at the end of the third week to check back in on goal progress. Texts were sent at the same time each morning for all participants.

Some reflect and prepare texts sent in the first 3 weeks offloaded effort from clinicians by providing information about guide‐line concordant SCP, and enhanced patient understanding and activation through the concept of providing spaced education (e.g., recommendations for each of the above topics, definition of each component of a SMART goal).^17^, ^18^, ^19^ Spaced education was provided on recommendations for each of the above topics (e.g., “Experts recommend that cancer survivors eat wisely, eating a diet high in vegetables, fruits and whole grains and low in sugars and fats”) and definition of each component of a SMART goal (e.g., “The S in SMART goals is for Specific. What exactly will you accomplish? Your health coach will partner with you to help you define your goal”). Some reflect and prepare texts included brief two‐way assessments to inform goal setting (e.g., following the above education on eating wisely, “I am satisfied with the food choices I make.” Reply with a “0” if you strongly disagree, “1” disagree, “2” neutral, “3” agree, “4” strongly agree) or served as cues for self‐reflection (e.g., to practice mindfulness and reflect on something discussed with the coach such as one's personal vision of optimal health).

The goal support text reminders and ratings sent after the second coaching call included some with a specific reference to one primary personalized goal entered into the system. For example, a message that followed up with a progress rating (“I am meeting my health goal.” Reply with a “0” if you strongly disagree, “1” disagree, “2” neutral, “3” agree, and “4” strongly agree) was sent weekly. Other goal support texts served as reminders to reflect on other content noted during the second coach call (e.g., “Reflect on your strengths that you wrote in your Guidebook. What has helped the most?”).

The eRefer portal tracked the number of participants e‐referred and our study team documented the number enrolled. Once enrolled onto the study, participants were asked to complete a baseline questionnaire. We collected all patient‐reported data remotely via a self‐administered REDCap or paper survey at baseline (before the first coaching call) and follow‐up (after the last coaching call). Intervention data were also collected through the above‐described text messages. Participants were offered compensation of up to $100 based on level of assessment completion. Study compensation was not mentioned by clinical staff who initially referred participants.

### 2.4 Measures

*Implementation processes*. Selection of implementation assessments was guided by the Conceptual Framework for Implementation Outcomes.^32^ Feasibility was primarily defined as the # participants enrolled/those e‐referred. Other indicators of feasibility were the number of participants enrolled per month, participant‐completed Feasibility of Intervention Measure (4‐items rated on a scale from 1 (*completely disagree*) to 5 (*completely agree*)),^33^ adherence rates, and retention rates. Adoption was assessed by the #e‐referred patients/those possible (based on an electronic chart review). Participants indicated whether they had viewed and thus engaged with the survivorship care plan document (yes/no/do not know). Adverse events related to the intervention were documented in a log for up to 30 days from the final intervention. Fidelity was assessed by patient adherence to text responses, adherence to coaching calls, length of coaching calls, and an observational assessment tool was completed for a subset of coaching calls.^25^ Each component was rated on a 3‐point scale (0 = Not yet competent, 1 = Competent, 2 = Proficient). Acceptability was measured by the participant‐completed Acceptability of Intervention Measure rated on a scale from 1 (*completely disagree*) to 5 (*completely agree*).^31^ Appropriateness was measured by the participant‐completed Intervention Appropriateness Measure rated on a scale from 1 (*completely disagree*) to 5 (*completely agree*).^31^ Participants also rated satisfaction with care as part of the study survey (Range: 1 = Very poor, 5 = Very good).

*Patient health outcomes* included proximal constructs of patient autonomy, assessed with the Index of Autonomous Functioning,^34^ self‐efficacy for managing cancer assessed with the Self‐efficacy to Manage Chronic Disease Scale,^35^, ^36^ mindful attention,^37^, ^38^ and health behaviors: tobacco use,^39^ alcohol use (alcohol use disorders identification test‐C higher scores indicate more hazardous alcohol use [range 0–12], men ≥4, women ≥3 is considered hazardous),^40^ physical activity (minutes/week, <90 min considered insufficient),^41^, ^42^ fruit and vegetable intake (mean for the responses were calculated in cups so ½ to 1 cup = 0.75 cups, at least 2–3 cups for both considered sufficient),^43^ and mindfulness meditation practice (In the last 30 days, how many days per week did you engage in a mindfulness meditation practice?). We also included outcomes assessed by the PROMIS Profile 29 version 2.0, ability to participate in social roles and activities, physical function, anxiety, depression, fatigue, sleep disturbance, pain, such that higher values indicate more of the construct measured (Range 0–100).^44^ General health was assessed with one‐item^45^ and cancer‐specific quality of life with the cancer‐specific subscale of the Quality of Life in Adult Cancer Survivors measure (cancer composite: financial problems, family distress, appearance problems, distress about recurrence, benefits of having cancer subscale, positive feelings subscale, higher scores indicate more problems or lower QOL).^46^, ^47^

*Clinical factors* abstracted from medical charts or self‐reported included cancer type, time since diagnosis, prior treatments, and comorbidities.

*Demographic characteristics* documented were age, sex, rural–urban residence (classified by the Rural–Urban Commuting Area codes [rural ≥4]^48^), race/ethnicity, marital status, education level, and limited or marginal health literacy (≤somewhat).^49^

### 2.5 Analyses

This pilot study had an accrual goal of 40 cancer survivors. The primary objective was to evaluate the feasibility of implementing SHARE‐S into clinical care, where feasibility was defined based on the rate of enrollment. Assuming a negative binomial distribution and true enrollment rate of 30%, the probability that we would have to approach 164 or more people to recruit 40 was <0.05. With this sample size of 40, we were able to estimate rates of interest within ±16% using exact 95% binomial confidence intervals. A sample size of 40 also allowed for reasonable estimates of SDs to be used to plan future studies.

We conducted descriptive statistics of data on other implementation processes, patient health outcomes, and background characteristics (i.e., clinical, demographic) to guide future study planning. The primary goal of the statistical analysis of these measures was to estimate mean differences with confidence intervals between time points along with Cohen's *d* effect sizes for use in future studies, not to perform formal hypothesis testing. We documented the number of patients seen in the Survivorship Clinic, the number e‐referred, and the percent who agreed to enroll in SHARE‐S. The 95% confidence intervals for participants who enrolled in SHARE‐S and for those who completed all assessments were calculated using the Clopper–Pearson Exact Method. We also tracked the frequency of any adverse events and percent of participants who completed the follow‐up visit to assess retention. An independent samples proportion test was used to compare the recruitment rate to the hypothesized value of 30%. Treatment fidelity was calculated as sum of total raw points earned divided by the points possible to create a standardized scaled score (to convert back on the rating scale of 0–2, the standardized scaled score was multiplied times two).^25^ For PROMIS outcomes, minimal within‐person change over time T‐score points were computed, with minimal important change (MIC) defined broadly for all PROMIS measures as ranging from 2.0 to 6.0.^50^ Cohen's d effect sizes are interpreted as: 0.2, small effect; 0.5, medium effect; and 0.8, large effect.^51^

## 3 RESULTS

### 3.1 Implementation processes

**FIGURE 1:**
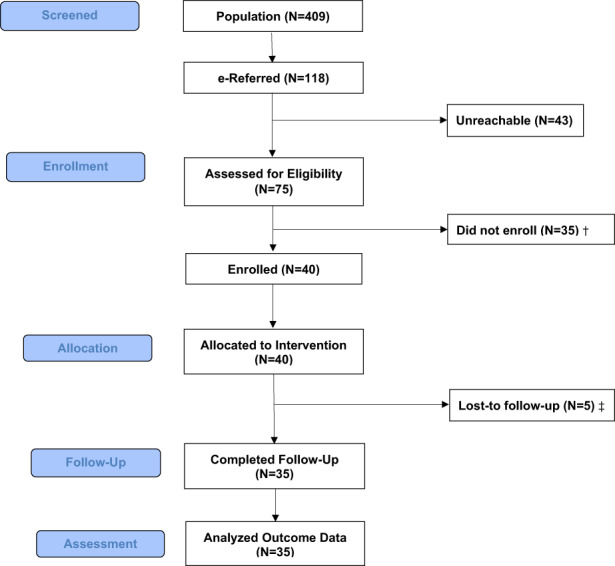
Study flow diagram. ^†^For those who were assessed foreligibility but did not enroll, two were not eligible (one did not havetext‐enabled phone and one expressed interest after study accrual was complete). Others declined participation (*N* = 33) because they were not interested in thestudy (*n* = 7); did not perceive need for the intervention (*n* = 3); were emotionally distressed or overwhelmed (*n* = 5); felt that the study requests were toodemanding (*n* = 10); had other health issues (*n* = 4); or unknown reasons (*n* = 4). ^‡^For those who were lost to follow‐up, four discontinued the intervention, and one did not complete the follow‐up assessment.

Characteristics of the referring clinics are described in Table 1. Figure 1 displays the study flow including the number of participants e‐referred, reasons for ineligibility, number of eligible patients approached, number and reasons for declining participation, and number enrolled. Regarding feasibility of recruitment, of the 118 patients referred over 17 months, from September 2020 through January 2022, we enrolled 40 (recruitment proportion = 34%, 95% confidence interval [CI]: 25%, 43%; observed proportion not significantly different than target of 30%, *p* = 0.54). The proportion of participants enrolled from the Comprehensive Cancer Center was 45% and 55% from the Affiliated Cancer Center. An average of seven participants were e‐referred per month (range: 0–17) and an average of two enrolled per month (range: 0–7). The proportion of adoption was 29% (# e‐referred patients/*n* = 409 in the clinics who were given a care plan, CI: 25%, 34%). The proportion of adoption by site was: Comprehensive Cancer Center = 19%; Affiliated Cancer Center = 55%. We incorporated iterative contextual modifications to refine clinic‐level implementation of SHARE‐S based on stakeholder feedback to increase feasibility. Notable adaptations included that one highly engaged referrer preferred to use the format of email rather than use the eRefer portal in order to provide more detail on the patient. We also adapted our approach to selecting the personnel who completed the referral. Initially this task was carried out by a clinic scheduler; we then expanded to include providers. Engagement with SCP document assessed at follow‐up was high: Yes = 97.1% (*N* = 33), I do not know = 2.9% (*N* = 1). No adverse events were reported.

**TABLE 1:** Characteristics of survivorship practice and demographics of new patients seen.

Survivorship practice characteristics	Comprehensive Cancer Center	Affiliated Cancer Center	Total (*N* = 602)
Number of total scheduled new patients (*N*)	435	167	602
Number of care plans given to patients (*N*)	297	112	409
Survivorship visit providers (*N*)	16	2	18
Survivorship visit format	In person	Telephone	

New patient demographics	*N* (%)	*N* (%)	Total (%)
Sex			
Female	243 (56%)	76 (46%)	320 (53%)
Male	192 (44%)	91 (54%)	282 (47%)
Race			
White	339 (78%)	118 (71%)	457 (76%)
Black	74 (17%)	36 (22%)	110 (18%)
Other	22 (5%)	13 (7%)	35 (6%)
Age (years)			
<30	57 (13%)	0 (0%)	57 (10%)
30–39	17 (4%)	2 (1%)	19 (3%)
40–49	30 (7%)	10 (6%)	41 (7%)
50–59	61 (14%)	20 (12%)	81 (13%)
60–69	135 (31%)	70 (42%)	205 (34%)
>70	135 (31%)	65 (39%)	199 (33%)

Cancer type	*N* (%)	*N* (%)	Total (%)
BMT/hematologic	91 (21%)	0 (0%)	91 (15%)
Lymphoma	61 (14%)	0 (0%)	61 (10%)
Pediatric	57 (13%)	0 (0%)	57 (9%)
Head and Neck	0 (0%)	9 (5%)	9 (2%)
Lung	109 (25%)	0 (0%)	109 (18%)
Breast	52 (12%)	74 (45%)	127 (21%)
Gastrointestinal	26 (6%)	0 (0%)	26 (4%)
Gynecologic	22 (5%)	0 (0%)	21 (4%)
Prostate	17 (4%)	84 (50%)	101 (17%)

### 3.2 Characteristics of participants enrolled

Those enrolled had a mean age of 57.4 years, 72.5% were female, 22.5% were Black or African American, and 12.5% were from a rural location (Table 2).

**TABLE 2:** Characteristics of e‐referred patients who agreed to participate in SHARE‐S (*N* = 40).

Characteristic	*N* (%)
Age (years)	
<30	5 (12.5%)
30–39	0 (0.0%)
40–49	4 (10.0%)
50–59	10 (25.0%)
60–69	12 (30.0%)
>70	9 (22.5%)
Sex	
Male	11 (27.5)
Female	29 (72.5)
Race	
White or Caucasian	29 (72.5)
Black or African American	9 (22.5)
Asian	1 (2.5)
Other	1 (2.5)
Hispanic or Latino (Yes)	1 (2.5)
Rural residence (Yes)	
Yes	5 (12.5)
No	35 (87.5)
Travel time to clinic (Minutes)—Mean (Standard deviation; SD)	31.5 (28.6)
Highest grade completed	
8th–11th grade	1 (2.5)
High School graduate or equivalent	5 (12.5)
Vocational or technical school/Associate's degree/some college	9 (22.5)
Bachelor's degree	17 (42.5)
Graduate or professional school	8 (20.0)
Marital status	
Currently married/living with partner	28 (70.0)
Separated/Divorced	4 (10.0)
Widowed	2 (5.0)
Single, never married	5 (12.5)
Prefer not to answer	1 (2.5)
Difficulty in paying monthly bills	
Very difficult	2 (5.0)
Somewhat difficult	8 (20.0)
Not very difficult/Not at all difficult	30 (75.0)
Number of times received medical income assistance	
Never	30 (75.0)
One–four times	8 (22.5)
More than four times	1 (2.5)
Confidence in filling out medical forms	
Extremely	27 (67.5)
Quite a bit	11 (27.5)
Somewhat	2 (5.0)
Used internet occasionally	
Yes	38 (95.0)
No	2 (5.0)
Primary tumor site	
Breast	18 (45.0)
Endometrial	3 (7.5)
Prostate	10 (25.0)
Other	9 (22.5)
History of cancer‐related surgical procedure	
Yes	25 (62.5)
No	15 (37.5)
History of radiation therapy	
Yes	28 (70.0)
No	12 (30.0)
History of chemotherapy	
Yes	18 (45.0)
No	22 (55.0)
Time since last surgery/treatment (Months)—Median (IQR)	5 (2, 39)
Body mass index—Mean (SD)	32.2 (6.8)

### 3.3 Participant engagement and perceptions of the SHARE‐S program

Once enrolled, 35 participants completed the follow‐up assessment (retention = 0.88; CI: 0.73, 0.96). Patient adherence to text responses, adherence to coaching calls, and length of coaching calls are summarized in Table 3a. Treatment fidelity ratings assessed by an observational tool^25^ reflected a score of 1.38 (between a competent score of 1 and proficient score of 2). Feasibility (*M* = 4.3, SD = 0.5, *n* = 34), acceptability (*M* = 4.4, SD = 0.6, *n* = 34), and appropriateness (*M* = 4.3, SD = 0.6, *n* = 34) were further supported by participant‐rated mean values higher than 4 (agree). Participants (*n* = 35) also reported high satisfaction with care (Mean = 4.8; SD = 0.4).

**TABLE 3:** Participant engagement in the SHARE‐S intervention over 6 weeks.

(a) Completion of intervention components	
Intervention components	Completion
	*N* (%)
*Coach call 1* (Week 0): Vision of optimal health, values, mindfulness, overview of survivorship care planning (SCP)	39 (98%)
Mean minutes (range)^a^	50 (13, 69)
*Reflect and prepare texts* (Weeks 0–3)^b^: Cues to reflect and practice mindfulness, SCP topics education and assessment of satisfaction, goal setting education—at least 1 of 8 requested responses per person	26 (65%)
*Coach call 2* (Week 3): Goal setting, action planning, problem solving, plan for self‐monitoring	36 (90%)
Goal documented in text message^b^	35 (88%)
Mean minutes (range)^a^	39 (26, 65)
*Goal support texts* (Weeks 3–6)^b^: Reminders of goals, values, strategies to overcome barriers, to monitor behavior, to enlist personal strengths, assessment of meeting goals—at least 1 of 3 requested responses per person	27 (68%)
Mean (SD) rating for: I am meeting my health goal (0—strongly disagree to 4—strongly agree)^b^	Week 4: 2.9 (0.9) Week 5: 2.8 (0.6) Week 6: 3.1 (0.8)
*Coach call 3* (Week 6): Review goals, plan for next steps to optimal health	36 (90%)
Mean minutes (range)^a^	28 (7, 48)

(b) Goal topics and select examples		
Number of participants (*N* = 36)^c^	Goal topics	Examples of specific goals
13 (36%)	Eat wisely	Sit down after coming home from work, at least 2 days a week, and plan next morning meal.
11 (31%)	Be physically active	Practice 30 minutes of yoga 2–3 times per week (Wednesday, Friday, 1 day on weekend) for 2 weeks to increase endurance.
1 (3%)	Be tobacco‐free/limit alcohol	Attend seven Al‐Anon meetings in 2 weeks.
0 (0%)	Strengthen social connections	Not applicable.
6 (17%)	Restore (e.g., Manage stress)	Meditate for 5 minutes before bedtime every night for 2 weeks.
1 (3%)	Get adequate rest	Turn off TV at 11:30 pm for 2 weeks to feel more rested.
0 (0%)	Engage in preventive care	Not applicable.
4 (11%)	Other personal development (e.g., Work)	To create a two‐pronged affairs in order/joy list in a journal that you will keep in your in‐tray.

^a^ Computed from length of audio recordings.

^b^ Computed using text messaging data.

^c^ One goal topic not entered in text message system assessed from coach call.

### 3.4 Goal setting

The most common primary goal topics self‐selected by participants were eat wisely (*n* = 13, 36%) and be physically active (*n* = 11, 31%; Table 3b). On average, participants progressed over time and agreed that they were meeting their goal with mean ratings of 3.1 (0 = strongly disagree to 4 = strongly agree) by Week 6 (Table 3a).

### 3.5 Effectiveness outcomes

Patient health outcomes are described in Table 4. Changes from baseline to follow‐up showed at least a small effect (Cohen's *d* = 0.2) for improvements in the following constructs: mindful attention, alcohol use, physical activity, fruit and vegetable intake, days of mindfulness practice, depressive symptoms, ability to participate in social roles and activities, cancer‐specific quality of life, benefits of having cancer, and positive feelings. The improvement in participants' ability to participate in social roles (e.g., I have trouble doing all of my usual work, my regular leisure activities with others) also demonstrated a minimally important difference.^50^

**TABLE 4:** Description of patient health outcomes.

Proximal outcomes (Possible range)	Baseline	Follow‐up	Difference	Cohen's *d* (95% CI)
	Mean (SD)	Mean (SD)	Mean (SD)	
	*N* = 40	*N* = 35	*N* = 35	
Index of autonomous functioning—autonomy (Range: 0–5)	4.3 (0.5)	4.3 (0.6)	0.07 (0.67)	0.11 (−0.23, −0.44)
Self‐efficacy for managing chronic disease scale (Range: 0–10)	7.7 (1.8)	7.8 (1.9)	0.13 (1.15)	0.12 (−0.22, 0.45)
Mindful attention awareness scale (Range: 0–6)	4.4 (1.3)	4.7 (1.3)	0.23 (1.13)	0.21 (−0.14, 0.54)

Health behaviors	*N* (%)	*N* (%)	*N* (%)	
	*N* = 40	*N* = 34	*N* = 34	
Tobacco use within last 7 days (Replied Yes)	1.0 (2.5)	1.0 (2.9)	0	
Alcohol use disorder (Mean, SD—Total audit C)	1.4 (1.9)	1.2 (1.6)	−0.32 (−0.58, −0.07)	−0.45 (−0.80, −0.09)
Physical activity (Mean, SD—Minutes/Week)	124.5 (140.0)	162.4 (157.2)	22.6 (114.8)	0.20 (−0.14, 0.54)
Fruit and vegetable intake (Mean, SD—Cups/Day)	2.7 (1.2)	3.2 (1.6)	0.58 (1.35)	0.43 (0.08, 0.78)
Mindfulness practice (Mean, SD—Days/Week)	1.8 (2.1)	3.4 (2.1)	1.53 (2.4)	0.64 (0.26, 1.00)

Health outcomes (Possible range)	Mean (SD)	Mean (SD)	Mean (SD)	
	*N* = 40	*N* = 35	*N* = 35	
PROMIS physical function (Range 0–100)	48.4 (8.2)	49.2 (8.5)	0.7 (4.1)	0.18 (−0.15, 0.52)
PROMIS anxiety (Range 0–100)	50.6 (9.8)	49.0 (8.5)	−1.1 (7.7)	−0.14 (−0.47, 0.19)
PROMIS depression (Range 0–100)	48.1 (9.3)	45.9 (7.7)	−1.2 (4.3)	−0.27 (−0.61, 0.07)
PROMIS fatigue (Range 0–100)	51.3 (10.9)	49.2 (11.5)	−1.4 (8.5)	−0.17 (−0.50, 0.17)
PROMIS sleep disturbance (Range 0–100)	49.6 (7.4)	48.9 (6.3)	−0.2 (5.8)	−0.04 (−0.37, 0.29)
PROMIS social roles (Range 0–100)	51.2 (9.4)	54.2 (10.8)	3.1 (6.5)	0.48 (0.12, 0.82)
PROMIS pain interference (Range 0–100)	51.5 (10.2)	50.3 (9.3)	−0.7 (4.6)	−0.15 (−0.48, 0.19)
PROMIS pain intensity (Range 0–10)	2.7 (2.4)	2.5 (2.4)	−0.11 (1.3)	−0.09 (−0.42, 0.24)
General health (Range: 1–5)	3.15 (0.83)	3.15 (0.89)	0.00 (0.6)	0.00 (−0.34, 0.34)
QLACS cancer‐specific Quality of Life (Range: 15–105), *n* = 34	47.0 (20.5)	40.9 (19.10)	−4.9 (13.2)	−0.37 (−0.72, −0.02)
QLACS benefits of having cancer (Range: 4–28)	13.9 (5.8)	12.2 (5.7)	−0.85 (4.2)	−0.20 (−0.14, 0.53)
QLACS positive feelings	10.6 (5.4)	9.5 (4.3)	−1.00 (3.5)	−0.29 (−0.06, 0.63)

*Note*: Higher values indicate more of each construct unless otherwise indicated (e.g., QLACS). CI=confidence interval. Patient‐Reported Outcomes Measurement Information System (PROMIS) values reported are standardized scores such that mean values generally center around 50 with standard deviations of 10. PROMIS health outcomes are from the Profile 29 version 2.0. Quality of Life in Adult Cancer Survivors (QLACS—higher scores indicate lower quality of life) and Survivorship Care Planning (SCP). AUDIT‐C = alcohol use disorders identification test.

To elaborate on changes in health behaviors described in Table 4, alcohol use decreased (*d* = −0.45) and the overall seven participants (20.6%; *n* = 34, men = 2, women = 5) who had hazardous alcohol use at baseline, two (5.9%; both women) improved to a non‐hazardous level. Fruit and vegetable intake also improved (*d* = 0.43). At baseline 34 (100%) participants were consuming less than recommended level of fruits and vegetables and 4 (11.8%) met recommended levels at follow‐up. Physical activity increased (*d* = 0.20), such that of the 14 (41.2%) participants at baseline (*n* = 34) who did not reach physical activity recommendations 6 (17.6%) changed to reach a recommended level of physical activity at follow‐up. Fifteen participants who did not practice meditation at baseline reported practicing at follow‐up.

## 4 DISCUSSION

This pilot study of the SHARE‐S implementation program shows promise for increasing patient engagement and is a first step toward addressing an evidence gap in implementing SCP to improve patient health outcomes. We successfully implemented SHARE‐S into clinical care and identified opportunities for improving future research. We also found promising results regarding effectiveness as assessed by patient health outcomes that warrant further evaluation.

Regarding our implementation focus, SHARE‐S successfully engaged cancer patients as demonstrated by the proportion of patients e‐referred who enrolled and the high adherence to the coaching program. The feasibility of e‐referral exceeded our a priori target. However, there was considerable variability in the number of e‐referrals from clinics per month and proportion of adoption by clinic site, reflecting the importance of highly engaged clinical referrers. Those who referred patients had mixed opinions regarding the eRefer portal. We learned from a particularly engaged referrer that it would be helpful to adapt the eRefer portal, so that it includes an optional text field that will allow further elaboration in future studies (e.g., preferred method and times of day for contacting patients). The clinical role of referrers and optimal timing of e‐referrals is likely to differ by local clinical context. E‐referrals to tobacco cessation programs have been successful^24^, ^52^ and this study showed potential for expanding the e‐referrals to programs that more broadly facilitate healthy lifestyles.

All of the patients who were highly adherent to the health self‐management coach calls set personalized health goals (*n* = 36; 90%). The most common general categories of goals selected were: Eat wisely (36%) and be physically active (31%). Examples of other specific goals included: to attend seven alcohol anonymous meetings and to meditate 5 minutes before bed. This coaching approach to facilitating patient‐driven personalized goals is likely to facilitate sustainable behavior change^53^, ^54^, ^55^ and be preferable to intervention approaches that direct participants to change a specific health behavior.

Assessment of success is challenging when providing the option for an infinite number of goals and we applied a novel approach of having participants rate the degree to which they were meeting their personal goal via weekly text messages. Furthermore, most participants actively engaged with the text message component that provided spaced education about SCP. Although there is evidence for the benefit of spaced education, this approach has rarely been used in the context of cancer and has not been applied to SCP.^17^, ^18^, ^19^

Those who participated in SHARE‐S showed promising improvements in health outcomes. There were small improvements in mindful attention, alcohol use, physical activity, fruit and vegetable intake, days of mindfulness practice, depressive symptoms, ability to participate in social roles and activities, cancer‐specific quality of life, benefits of having cancer, and positive feelings. The improvement in participants' ability to participate in social roles also demonstrated a minimally important difference.^50^ These results are consistent with other SCP studies that used shared decision making and led to improvements in social functioning,^9^ as well as other health outcomes (e.g., depressive symptoms, emotional and physical functioning, quality of life).^9^, ^11^ It was interesting that over a third of participants reported a new mindfulness practice and there was an overall increase in mindful attention after mindfulness was introduced as a component of the health coaching sessions. Mindfulness is a foundational practice for becoming aware of discrepancies between one's current and desired states and thus motivating autonomous engagement in self‐regulation.^14^, ^56^

### 4.1 Clinical implications

Supporting patients to increasingly engage in self‐management is an important component of proposed systematic changes that involve risk stratifying delivery of follow‐up cancer care.^57^ Participants in SHARE‐S were open to working with a health coach and implemented positive health behaviors including mindfulness, which is not a standard offering from medical providers. Thus, in addition to assisting in self‐management of healthy survivorship behaviors, health coaches offer complementary tools that teach patients how to successfully engage in a self‐management process generalizable to supporting ongoing healthy living.^14^ Health coaching is an emerging field such that the American Medical Association Current Procedural Terminology (CPT) panel launched a relevant new level 3 CPT© for health and wellness coaching.^58^ Others have also proposed the benefit of incorporating a health coach (or health promotionist) with added experience in using digital technologies, into cancer survivorship clinics to provide a multiple‐behavior change intervention that would address the gap in addressing common behavioral risk factors.^59^

### 4.2 Study limitations and future directions

A limitation of this study was that we did not assess demographic characteristics of those e‐referred to the study. Thus, a future study may more clearly examine if participants enrolled in our program were more likely to be female because of a bias in clinician referrals or difference in patients' likelihood of agreeing to participate. Also, it is a limitation that we do not know if our sample was representative regarding how many participated from a rural location since our available data on rurality were from the broader health system and not specific to the clinics that participated in our study. Furthermore, our assessment of implementation processes was not comprehensive. It will be important to assess other provider and organizational‐level implementation processes (e.g., implementation cost, penetration, sustainability)^32^ in a future study with additional clinics. For example, future research could collect data on healthcare utilization related to receiving and following up on referrals for other appropriate supportive services. Additionally future research could potentially strengthen the intervention by considering a more systematic approach to selecting strategies for improving survivorship care planning such as implementation mapping.^60^

## 5 CONCLUSIONS

In summary, we successfully implemented SHARE‐S as demonstrated by a high level of participant engagement. We were iteratively responsive to our collaborators' feedback as we delivered SHARE‐S and adapted components to improve the program for future studies. Some adaptations will change the tools (e.g., adding an open‐text field to eRefer) and others may continue to be modified based on the local context (e.g., how e‐referrals fit within the clinical flow). Future studies will also more clearly document how systematically clinicians provide e‐referrals and other provider and organizational‐level implementation processes across more clinics. Those who participated in SHARE‐S also showed promising improvements in health outcomes and the improvement in participants' ability to participate in social roles was clinically important. Thus, SHARE‐S provides a promising solution to supporting patients to engage in self‐management, which may improve health outcomes in follow‐up cancer care.

## AUTHOR CONTRIBUTIONS

**Stephanie Jean Sohl:** Conceptualization (equal); data curation (equal); methodology (equal); project administration (lead); resources (equal); supervision (equal); visualization (equal); writing – original draft (lead). **Rajani S. Sadasivam:** Conceptualization (supporting); funding acquisition (supporting); methodology (supporting); writing – review and editing (supporting). **Carol Kittel:** Data curation (equal); formal analysis (lead); software (supporting); visualization (supporting); writing – original draft (supporting). **Emily Dressler:** Conceptualization (supporting); data curation (supporting); formal analysis (supporting); funding acquisition (supporting); methodology (supporting); software (equal); supervision (supporting); visualization (equal); writing – review and editing (supporting). **Stacy Wentworth:** Conceptualization (supporting); methodology (supporting); writing – review and editing (supporting). **Kavitha Balakrishnan:** Conceptualization (supporting); methodology (supporting); writing – review and editing (supporting). **Kathryn E Weaver:** Conceptualization (supporting); writing – review and editing (supporting). **Rebecca A. Dellinger:** Data curation (equal); project administration (equal); writing – original draft (supporting). **Nicole Puccinelli‐Ortega:** Conceptualization (equal); project administration (equal); writing – review and editing (supporting). **Sarah L. Cutrona:** Conceptualization (supporting); funding acquisition (equal); writing – review and editing (supporting). **Kristie L. Foley:** Conceptualization (supporting); funding acquisition (equal); writing – review and editing (supporting). **Thomas Houston:** Conceptualization (equal); data curation (equal); funding acquisition (equal); methodology (equal); resources (equal); supervision (lead); visualization (equal); writing – review and editing (equal).

## FUNDING INFORMATION

This research was supported by the National Cancer Institute as a pilot study component of Grant Award Number P50CA244693 (Foley/Houston/Cutrona, Multi‐P.I.s) iDAPT (Implementation & Informatics: Developing Adaptable Processes and Technologies for Cancer Control), which is one of six National Cancer Institute‐funded Implementation Science Centers for Cancer Control and through the Comprehensive Cancer Center of Wake Forest University Cancer Center Support Grant (P30CA012197). The content is solely the responsibility of the authors and does not necessarily represent the official views of the NIH. In addition, the views expressed in this paper are those of the authors and do not necessarily reflect the position or policy of the Department of Veterans Affairs or the United States government.

## CONFLICT OF INTEREST STATEMENT

The authors report there are no competing interests to declare.

## ETHICS APPROVAL AND CONSENT TO PARTICIPATE

This trial was approved by the local Institutional Review Board and informed and performed in accordance with the ethical standards as laid down in the 1964 Declaration of Helsinki and its later amendments or comparable ethical standards. Consent was obtained from all individual participants included in the study.

## Supporting information


Table S1

## Data Availability

The data that support the findings of this study are available from the corresponding author upon reasonable request.

## References

[1] American Cancer Society . Cancer Treatment & Survivorship Facts & Figures 2019–2021. American Cancer Society; 2019.

[2] McCorkle R , Ercolano E , Lazenby M , et al. Self‐management: enabling and empowering patients living with cancer as a chronic illness. Cancer. 2011;61(1):50‐62. PMID:21205833. 10.3322/caac.20093 PMC3058905 21205833

[3] National Research Council . From Cancer Patient to Cancer Survivor: Lost in Transition. The National Academies Press; 2005. ISBN:0‐309‐09595‐6.

[4] Jacobsen PB , DeRosa AP , Henderson TO , et al. Systematic review of the impact of cancer survivorship care plans on health outcomes and health care delivery. J Clin Oncol. 2018;36(20):2088‐2100. PMID:29775389. 29775389 10.1200/JCO.2018.77.7482 PMC6036622

[5] Birken SA , Urquhart R , Munoz‐Plaza C , et al. Survivorship care plans: are randomized controlled trials assessing outcomes that are relevant to stakeholders? J Cancer Surviv Res Pract. 2018;12(4):495‐508. PMID:29572602. 10.1007/s11764-018-0688-6 PMC6054562 29572602

[6] van de Poll‐Franse LV , Nicolaije KAH , Ezendam NPM . The impact of cancer survivorship care plans on patient and health care provider outcomes: a current perspective. Acta Oncol. 2017;56(2):134‐138. PMID:28084140. 28084140 10.1080/0284186X.2016.1266080

[7] Kline RM , Arora NK , Bradley CJ , et al. Long‐term survivorship care after cancer treatment—summary of a 2017 National Cancer Policy Forum Workshop. J Natl Cancer Inst. 2018;110(12):1300‐1310. doi:10.1093/jnci/djy176 30496448 PMC6658871

[8] Keesing S , McNamara B , Rosenwax L . Cancer survivors' experiences of using survivorship care plans: a systematic review of qualitative studies. J Cancer Surviv Res Pract. 2015;9(2):260‐268. PMID:25343971. 10.1007/s11764-014-0407-x PMC4441735 25343971

[9] Kvale EA , Huang C‐HS , Meneses KM , et al. Patient‐centered support in the survivorship care transition: outcomes from the patient‐owned survivorship care plan intervention. Cancer. 2016;122(20):3232‐3242. PMID:27387096. 27387096 10.1002/cncr.30136

[10] Selove R , Birken SA , Skolarus TA , Hahn EE , Sales A , Proctor EK . Using implementation science to examine the impact of cancer survivorship care plans. J Clin Oncol. 2016;34(32):3834‐3837. doi:10.1200/JCO.2016.67.8060 27621409 PMC5477985

[11] Reb A , Ruel N , Fakih M , et al. Empowering survivors after colorectal and lung cancer treatment: pilot study of a self‐management survivorship care planning intervention. Eur J Oncol Nurs. 2017;29:125‐134. PMID:28720259. 28720259 10.1016/j.ejon.2017.06.003 PMC5539921

[12] Bodenheimer T , Wagner EH , Grumbach K . Improving primary care for patients with chronic illness. JAMA. 2002;288(14):1775‐1779. PMID:12365965. 12365965 10.1001/jama.288.14.1775

[13] Gee PM , Greenwood DA , Paterniti DA , Ward D , Miller LMS . The eHealth enhanced chronic care model: a theory derivation approach. J Med Internet Res. 2015;17(4):e86. doi:10.2196/jmir.4067 25842005 PMC4398883

[14] Sohl SJ , Birdee G , Elam R . Complementary tools to empower and sustain behavior change: motivational interviewing and mindfulness. Am J Lifestyle Med. 2016;10(6):429‐436. PMID:28239308. 28239308 10.1177/1559827615571524 PMC5319432

[15] Birken SA , Mayer DK , Weiner BJ . Survivorship care plans: prevalence and barriers to use. J Cancer Educ. 2013;28(2):290‐296. PMID:23526552. 23526552 10.1007/s13187-013-0469-x PMC3665729

[16] Committee on Quality of Health Care in America, Institute of Medicine . Crossing the Quality Chasm: A New Health System for the 21st Century. National Academy Press. Accessed September 19, 2013. http://www.nap.edu/openbook.php?record_id=10027&page=4

[17] Kerfoot BP . Learning benefits of on‐line spaced education persist for 2 years. J Urol. 2009;181(6):2671‐2673. PMID:19375095. 19375095 10.1016/j.juro.2009.02.024

[18] Kerfoot BP , Lawler EV , Sokolovskaya G , Gagnon D , Conlin PR . Durable improvements in prostate cancer screening from online spaced education a randomized controlled trial. Am J Prev Med. 2010;39(5):472‐478. PMID:20965387. 20965387 10.1016/j.amepre.2010.07.016 PMC2994103

[19] Kerfoot BP , Kearney MC , Connelly D , Ritchey ML . Interactive spaced education to assess and improve knowledge of clinical practice guidelines: a randomized controlled trial. Ann Surg. 2009;249(5):744‐749. PMID:19387336. 19387336 10.1097/SLA.0b013e31819f6db8

[20] Earle C . Failing to plan is planning to fail: improving the quality of care with survivorship care plans. J Clin Oncol. 2006;24(32):5112‐5116. PMID:17093272. 17093272 10.1200/JCO.2006.06.5284

[21] National Comprehensive Cancer Network (NCCN) . NCCN Guidelines: Survivorship. 2019. Report No.: Version 2.2019.

[22] McCreight MS , Rabin BA , Glasgow RE , et al. Using the practical, robust implementation and sustainability model (PRISM) to qualitatively assess multilevel contextual factors to help plan, implement, evaluate, and disseminate health services programs. Transl Behav Med. 2019;9(6):1002‐1011. doi:10.1093/tbm/ibz085 31170296

[23] Miller CJ , Barnett ML , Baumann AA , Gutner CA , Wiltsey‐Stirman S . The FRAME‐IS: a framework for documenting modifications to implementation strategies in healthcare. Implement Sci. 2021;16(1):36. doi:10.1186/s13012-021-01105-3 33827716 PMC8024675

[24] Houston TK , Sadasivam RS , Allison JJ , et al. Evaluating the QUIT‐PRIMO clinical practice ePortal to increase smoker engagement with online cessation interventions: a national hybrid type 2 implementation study. Implement Sci IS. 2015;10:154. PMID:26525410. 26525410 10.1186/s13012-015-0336-8 PMC4630887

[25] Sohl SJ , Lee D , Davidson H , et al. Development of an observational tool to assess health coaching fidelity. Patient Educ Couns. 2020;104:642‐648. doi:10.1016/j.pec.2020.08.040 32948400 PMC8942015

[26] Powell BJ , Waltz TJ , Chinman MJ , et al. A refined compilation of implementation strategies: results from the expert recommendations for implementing change (ERIC) project. Implement Sci. 2015;10(1):21. doi:10.1186/s13012-015-0209-1 25889199 PMC4328074

[27] Quintiliani LM , Foster M , Oshry LJ . Preferences of mHealth app features for weight management among breast cancer survivors from underserved populations. Psychooncology. 2019;28(10):2101‐2104. doi:10.1002/pon.5190 31368197 PMC6786913

[28] Michie S , Wood CE , Johnston M , Abraham C , Francis JJ , Hardeman W . Behaviour change techniques: the development and evaluation of a taxonomic method for reporting and describing behaviour change interventions (a suite of five studies involving consensus methods, randomised controlled trials and analysis of qualitative data). Health Technol Assess Winch Engl. 2015;19(99):1‐188. PMID:26616119. 10.3310/hta19990 PMC4781650 26616119

[29] Michie S , Johnston M , Abraham C , Lawton R , Parker D , Walker A . “Psychological theory” group. Making psychological theory useful for implementing evidence based practice: a consensus approach. Qual Saf Health Care. 2005;14(1):26‐33. PMID:15692000. 15692000 10.1136/qshc.2004.011155 PMC1743963

[30] Damschroder LJ , Reardon CM , Sperber N , Robinson CH , Fickel JJ , Oddone EZ . Implementation evaluation of the telephone lifestyle coaching (TLC) program: organizational factors associated with successful implementation. Transl Behav Med. 2017;7(2):233‐241. PMID:27688249. 27688249 10.1007/s13142-016-0424-6 PMC5526796

[31] Sohl SJ , Befus D , Tooze JA , et al. Feasibility of systems support mapping to guide patient‐driven self‐management in colorectal cancer survivors. Psychol Health. 2021;1‐21. 10.1080/08870446.2021.1979549 PMC8957632 34570677

[32] Proctor E , Silmere H , Raghavan R , et al. Outcomes for implementation research: conceptual distinctions, measurement challenges, and research agenda. Adm Policy Ment Health. 2011;38(2):65‐76. doi:10.1007/s10488-010-0319-7 20957426 PMC3068522

[33] Weiner BJ , Lewis CC , Stanick C , et al. Psychometric assessment of three newly developed implementation outcome measures. Implement Sci. 2017;12(1):108. PMID:28851459. 28851459 10.1186/s13012-017-0635-3 PMC5576104

[34] Weinstein N , Przybylski AK , Ryan RM . The index of autonomous functioning: development of a scale of human autonomy. J Res Personal. 2012;46(4):397‐413. doi:10.1016/j.jrp.2012.03.007

[35] Ritter PL , Lorig K . The English and Spanish self‐efficacy to manage chronic disease scale measures were validated using multiple studies. J Clin Epidemiol. 2014;67(11):1265‐1273. doi:10.1016/j.jclinepi.2014.06.009 25091546

[36] Moore SM , Schiffman R , Waldrop‐Valverde D , et al. Recommendations of common data elements to advance the science of self‐Management of Chronic Conditions. J Nurs Scholarsh. 2016;48(5):437‐447. PMID:27486851. 27486851 10.1111/jnu.12233 PMC5490657

[37] Brown KW , Ryan RM . The benefits of being present: mindfulness and its role in psychological well‐being. J Pers Soc Psychol. 2003;84(4):822‐848. doi:10.1037/0022-3514.84.4.822 12703651

[38] Osman A , Lamis DA , Bagge CL , Freedenthal S , Barnes SM . The mindful attention awareness scale: further examination of dimensionality, reliability, and concurrent validity estimates. J Pers Assess. 2016;98(2):189‐199. PMID:26560259. 26560259 10.1080/00223891.2015.1095761

[39] NCI‐AACR Cancer Patient Tobacco Use Assessment Task Force . Cancer Patient Tobacco Use Questionnaire (C‐TUQ). 2016. https://cancercontrol.cancer.gov/brp/research/

[40] Bush K , Kivlahan DR , McDonell MB , Fihn SD , Bradley KA . The AUDIT alcohol consumption questions (AUDIT‐C): an effective brief screening test for problem drinking. Ambulatory care quality improvement project (ACQUIP). Alcohol use disorders identification test. Arch Intern Med. 1998;158(16):1789‐1795. PMID:9738608. 9738608 10.1001/archinte.158.16.1789

[41] Coleman KJ , Ngor E , Reynolds K , et al. Initial validation of an exercise “vital sign” in electronic medical records. Med Sci Sports Exerc. 2012;44(11):2071‐2076. PMID:22688832. 22688832 10.1249/MSS.0b013e3182630ec1

[42] Centers for Medicare and Medicaid Services . The Accountable Health Communities Health‐Related Social Needs Screening Tool. Center for Medicare and Medicaid Innovation. Accessed May 5, 2022. https://innovation.cms.gov/files/worksheets/ahcm‐screeningtool.pdf

[43] Yaroch AL , Tooze J , Thompson FE , et al. Evaluation of three short dietary instruments to assess fruit and vegetable intake: the National Cancer Institute's food attitudes and behaviors survey. J Acad Nutr Diet. 2012;112(10):1570‐1577. PMID:23017567. 23017567 10.1016/j.jand.2012.06.002 PMC3775662

[44] Cella D , Riley W , Stone A , et al. The patient‐reported outcomes measurement information system (PROMIS) developed and tested its first wave of adult self‐reported health outcome item banks: 2005–2008. J Clin Epidemiol. 2010;63(11):1179‐1194. PMID:20685078. 20685078 10.1016/j.jclinepi.2010.04.011 PMC2965562

[45] The Mos Short‐Form General Health Survey . Single Item Vs Multiple Measures of Health‐Related Quality of Life: some Nuances—G. I. J. M. Kempen; 1992. Accessed February 27, 2020. doi:10.2466/pr0.1992.70.2.608 1598378

[46] Avis NE , Smith KW , McGraw S , Smith RG , Petronis VM , Carver CS . Assessing quality of life in adult cancer survivors (QLACS). Qual Life Res. 2005;14(4):1007‐1023. PMID:16041897. 16041897 10.1007/s11136-004-2147-2

[47] Sohl SJ , Levine B , Avis NE . Evaluation of the quality of life in adult cancer survivors (QLACS) scale for early post‐treatment breast cancer survivors. Qual Life Res Int J Qual Life Asp Treat Care Rehabil. 2015;24(1):205‐212. PMID:24996392. 10.1007/s11136-014-0749-x PMC4282954 24996392

[48] Rural Urban Commuting Area Codes . USDA Econ Res Serv. Accessed October 12, 2017. https://www.ers.usda.gov/data‐products/rural‐urban‐commuting‐area‐codes/

[49] Wallace LS , Rogers ES , Roskos SE , Holiday DB , Weiss BD . Brief report: screening items to identify patients with limited health literacy skills. J Gen Intern Med. 2006;21(8):874‐877. PMID:16881950. 16881950 10.1111/j.1525-1497.2006.00532.x PMC1831582

[50] Terwee CB , Peipert JD , Chapman R , et al. Minimal important change (MIC): a conceptual clarification and systematic review of MIC estimates of PROMIS measures. Qual Life Res Int J Qual Life Asp Treat Care Rehabil. 2021;30(10):2729‐2754. PMID:34247326. 10.1007/s11136-021-02925-y PMC8481206 34247326

[51] Cohen J . Statistical Power Analysis for the Behavioral Sciences. 2nd ed. Lawrence Erlbaum Associates; 1988.

[52] Sadasivam RS , Hogan TP , Volkman JE , et al. Implementing point of care “e‐referrals” in 137 clinics to increase access to a quit smoking internet system: the quit‐Primo and National Dental PBRN HI‐QUIT studies. Transl. Behav Med. 2013;3(4):370‐378. PMID:24294325. 10.1007/s13142-013-0230-3 PMC3830021 24294325

[53] Deci EL , Ryan RM . Self‐determination theory: a macrotheory of human motivation, development, and health. Can Psychol. 2008;49(3):182‐185. doi:10.1037/a0012801

[54] Ng JYY , Ntoumanis N , Thøgersen‐Ntoumani C , et al. Self‐determination theory applied to health contexts a meta‐analysis. Perspect Psychol Sci. 2012;7(4):325‐340. doi:10.1177/1745691612447309 26168470

[55] Mann T , de Ridder D , Fujita K . Self‐regulation of health behavior: social psychological approaches to goal setting and goal striving. Health Psychol. 2013;32(5):487‐498. doi:10.1037/a0028533 23646832

[56] Brown KW , Ryan RM , Creswell JD . Mindfulness: theoretical foundations and evidence for its salutary effects. Psychol Inq. 2007;18(4):211‐237. doi:10.1080/10478400701598298

[57] Mayer DK , Alfano CM . Personalized risk‐stratified cancer follow‐up care: its potential for healthier survivors, happier clinicians, and lower costs. J Natl Cancer Inst. 2019;111(5):442‐448. doi:10.1093/jnci/djy232 30726949 PMC6804411

[58] The National Board for Health and Wellness Coaching (NBHWC) . American Medical Association Approves New Category III CPT Codes for Coaching. 24–7 Press Release 2019. Accessed February 3, 2020. https://www.24‐7pressrelease.com/press‐release/466893/american‐medical‐association‐approves‐new‐category‐iii‐cpt‐codes‐for‐coaching

[59] Spring B , Stump T , Penedo F , Pfammatter AF , Robinson JK . Toward a health‐promoting system for cancer survivors: patient and provider multiple behavior change. Health Psychol. 2019;38(9):840‐850. PMID:31436465. 31436465 10.1037/hea0000760 PMC6709684

[60] Fernandez ME , ten Hoor GA , van Lieshout S , et al. Implementation mapping: using intervention mapping to develop implementation strategies. Front Public Health. 2019;7:7. doi:10.3389/fpubh.2019.00158 31275915 PMC6592155

