# Supplementary material for: Pilot study of implementing the Shared Healthcare Actions & Reflections Electronic systems in Survivorship (SHARE‐S) program in coordination with clinical care
Source: Cancer Med. 2023 Apr 25;12(11):12847–60. doi: 10.1002/cam4.5965 (PMC10278506; doi:10.1002/cam4.5965)
Supplement: Supplementary file 1 — Table S1 [file CAM4-12-12847-s001.docx]

| **Supporting Information**  **Supplemental Table.** Example intervention modifications resulting from stakeholder feedback | |
| --- | --- |
| **Health Coach Feedback** | **Resulting Intervention Modifications** |
| Connect the daily text messages back to the coaching sessions and to each other. | We created text messages that linked to content completed in the Personal Health Journey Guidebook during coaching sessions. For example: “Reflect on your strengths noted in your Guidebook. What did you identify that will help you to achieve your goal? Write down any others that may come to mind.” |
| Rate level of progress for goal evaluation 1-10 during post-visit messages. | Included ratings of goal progress in goal support messages: Please rate the following: “I am meeting my health goal.” Reply with a “0” if you strongly disagree, “1” disagree, “2” neutral, “3” agree and “4” strongly agree. |
| Include mindfulness exercises | Mindfulness practices were added to messages: “Reflect: If it feels comfortable, take a few moments for MINDFULNESS. Refer to the Guidebook for a mindfulness practice.” |
| Include SMART goals definitions | SMART goal definitions were incorporated in messages: “The M in SMART goals is for Measureable. How will you know when you have reached your goal? Think about it for now and your health coach will work with you to clarify when you will know that you have achieved your goal.” |
| **Healthcare Provider Feedback** | **Resulting Modifications** |
| Offer the provider team in-person training about the program during their monthly meeting as well as a reminder one-sheet document. | We presented to the provider team and provided a one-sheet summary document. |
| Provide patients with a mindfulness definition in the wellness guide. | We added a mindfulness definition and example practice in the Guidebook. |
| One provider said she would be in favor of having this intervention start after the first survivorship visit because some patients may not be able to engage fully prior to knowing they don’t have cancer recurrence. | We modified the inclusion criteria to also allow for referrals and intervention delivery after any survivorship visit. |
| **Cancer Survivor Feedback** | **Resulting Modifications** |
| Be flexible with the time messages are sent. Some patients work and some are unable to or forget to reply. | Participants were able to reply to text messages, if applicable, at any time before the next one was sent. |
| Concerned about patient replying to unmonitored text message with an urgent need. | Added to welcome message: “Some messages may ask for you to reply. Your replies are not monitored, so please contact the study team or your healthcare providers with any questions.” |
